# 

**Published:** 1883-09

**Authors:** 


ORIGINAL COMMUNICATIONS:

The Application of Nitrous Oxide and Air, or Nitrous Oxide and Oxygen, under Pressure, to Produce Anæsthesia in Persons for Dental and Surgical Operations. Dr. E. P. Howland ........ 455
Æsthetic Dentistry. Dr. E. H. Bunting ........ 460
Filling Teeth and the Philosophy Thereof. Dr. J. Hayhurst ........ 465

ORIGINAL TRANSLATIONS AND ABSTRACTS:

Histological Investigation upon the Action of Arsenious Acids on the Nerve Fibres of the Pulp and the Dentinal Tissues. Dr. C. F. W. Bodecker ........ 468
Good for Mexico ........ 470
Product of the Hen ........ 471
Definitions ........ 471

REPORTS OF SOCIETY MEETINGS:

New Jersey State Dental Society ........ 472
American Dental Association ........ 489
The American Dental Society of Europe ........ 495

EDITORIAL:

The American Dental Association ........ 497
The Pearl in the Oyster ........ 501
Correction ........ 501
To Subscribers ........ 502

NEW APPLIANCES AND MATERIALS:

Enamel Chisels ........ 502
Impression Cups ........ 502
Listerine ........ 503

BIBLIOGRAPHICAL:

Microscopical Diagnosis ........ 503

CURRENT NEWS AND OPINION:

American Association for the Advancement of Science ........ 504
American Society of Microscopists ........ 505
Novel Laryngoscope ........ 506
New College ........ 506
Transactions of the Dental Society of the State of New York ........ 507
Painting Teeth ........ 507
Dental Legislation ........ 507
Resigned ........ 507
New Book ........ 508
The Ohio Journal ........ 508
Editorial Change ........ 508
Called ........ 508

SELECTIONS:

Ozone as Anæsthetic and Hypnotic ........ 508
The Life of Man ........ 510
Nerve Instruments ........ 510
Freckles ........ 510
Notched Teeth ........ 511

